# Extracellular vesicle concentrations of glial fibrillary acidic protein and neurofilament light measured 1 year after traumatic brain injury

**DOI:** 10.1038/s41598-021-82875-0

**Published:** 2021-02-16

**Authors:** Spencer Flynn, Jacqueline Leete, Pashtun Shahim, Cassandra Pattinson, Vivian A. Guedes, Chen Lai, Christina Devoto, Bao-Xi Qu, Kisha Greer, Brian Moore, Andre van der Merwe, Vindhya Ekanayake, Jessica Gill, Leighton Chan

**Affiliations:** 1 Rehabilitation Medicine Department, National Institutes of Health Clinical Center, Bethesda, MD USA; 2 National Institute of Nursing Research, National Institutes of Health, Bethesda, MD USA; 3 Center for Neuroscience and Regenerative Medicine, Bethesda, MD USA; 4 The Henry M. Jackson Foundation for the Advancement of Military Medicine, Bethesda, MD USA

**Keywords:** Brain injuries, Biomarkers

## Abstract

Traumatic brain injury (TBI) is linked to long-term symptoms in a sub-set of patients who sustain an injury, but this risk is not universal, leading us and others to question the nature of individual variability in recovery trajectories. Extracellular vesicles (EVs) are a promising, novel avenue to identify blood-based biomarkers for TBI. Here, our aim was to determine if glial fibrillary acidic protein (GFAP) and neurofilament light (NfL) measured 1-year postinjury in EVs could distinguish patients from controls, and whether these biomarkers relate to TBI severity or recovery outcomes. EV GFAP and EV NfL were measured using an ultrasensitive assay in 72 TBI patients and 20 controls. EV GFAP concentrations were elevated in moderate and severe TBI compared to controls (*p*’s < 0.001) and could distinguish controls from moderate (AUC = 0.86) or severe TBI (AUC = 0.88). Increased EV GFAP and EV NfL levels were associated with lower 1-year Glasgow Outcome Scale–Extended (GOS-E) score (*p*’s < 0.05). These findings suggest that blood-derived EV concentrations of GFAP and NfL drawn even 1 year after injury are higher in TBI patients compared to controls, and are related to injury severity and poor recovery outcomes, suggesting that TBIs alter the activity of these biomarkers, likely contributing to individual variability in recovery.

## Introduction

Traumatic brain injury (TBI) is a heterogeneous condition with highly variable outcomes, with some data suggesting that the severity of the TBI relates to risk, yet this is not conclusive. Previous studies show that some TBI patients experience marked recoveries over time, while others report poor recovery or declines^1–3^. In patients who experience chronic sequela, many report that their symptoms profoundly impact quality of life^4^. It is imperative that efficient and targeted tests are developed that can aid in identifying patients with a TBI as well as those at risk for lasting symptoms.

Blood-based biomarkers may provide the ability to diagnose TBI and to predict patient outcomes following a TBI. Numerous studies have assessed the utility of serum or plasma glial fibrillary acidic protein (GFAP)—an astrocytic cytoskeleton protein released after astrocytic cell death—and neurofilament light (NfL)—an axonal protein associated with damaged axons—as diagnostic and prognostic markers of TBI^5–11^. Recently, another avenue for TBI biomarker research has been investigated: quantifying proteins contained in extracellular vesicles (EVs). EVs exist in nearly all eukaryotic fluids, carry cargo, including proteins, to and from all areas in the body, and can protect their contents from degradation by proteases or ribonucleases that are common in blood^12,13^. EVs are suggested to be more biologically active than proteins found within circulating blood^14^. Moreover, EVs can transverse the blood–brain barrier and can be isolated from peripheral biofluids, making them a promising new avenue of investigation for central nervous system functioning and for the development of TBI biomarkers^13,15^. Our lab has recently investigated the utility of EV biomarkers in a sample of military personnel and Veterans with remote mTBI. Our findings suggest that repetitive, remote mTBIs are associated with elevations of EV NfL, especially among individuals with chronic neurobehavioral or psychological symptoms^9^. However, the utility of EV NfL in civilian patients with a history of single TBI and in comparison with EV GFAP has not been previously investigated.

In this study, we used an ultrasensitive assay to examine the ability of EV levels of GFAP and NfL to identify patients with a TBI (ranging from mild to severe) 1 year postinjury, and to determine associations with recovery outcomes 1–5 years postinjury. We hypothesized that NfL and GFAP would be detectable in EVs at 1 year after a single TBI, and higher EV concentrations would correlate with injury severity and functional outcome.

## Results

### Demographic and clinical characteristics

Participants included for analysis were 71 TBI patients and 20 healthy volunteers. They were predominantly white (74.7%) and male (62.6%) with an age range of 19–80 years and a mean of 45 years (Table 1). There were 27 mild TBI (mTBI) patients, 29 moderate TBI (modTBI) patients, and 15 severe TBI (sTBI) patients. Within the TBI patients, there were high rates of depression and anxiety diagnoses as well as use of psychoactive drugs (Table 1). Age and sex did not differ significantly between the TBI and control groups. The mean time between outcome tests at 1 year postinjury to the final outcome test was 3 years (SD 1.5). There were no differences in race, age, sex, or mechanism of injury between the TBI severity groups.

**Table 1:** Demographics, clinical characteristics, and biomarker concentrations at 1 year.

	TBI (n = 71)	HV (n = 20)	*p*	F/${\upchi }^{2}$
Age in years, mean (SD)	47.6 (17.4)	42.9 (13.6)	0.265	1.26
Sex, female:male	23:48	11:9	0.113	2.51
**Race, no. (%)**				
White	57 (80)	11 (55)	0.009	11.57
African American	7 (10)	8 (40)		
Asian	2 (3)	1 (5)		
Multiple races	5 (7)	0 (0)		
**Injury severity, no. (%)**				
Mild	27 (38)	–	–	–
Moderate	29 (41)	–	–	–
Severe	15 (21)	–	–	–
**Mechanism of injury, no. (%)**				
Acceleration/deceleration	26 (37)	–	–	–
Direct impact	20 (28)	–	–	–
Fall	25 (35)	–	–	–
Blast	0 (0)	–	–	–
Prior military service, no (%)	0 (0)	0 (0)	NA	NA
**Prior psychiatric history, no. (%)**				
Anxiety	7 (10)	–	–	–
Depression	14 (20)	–	–	–
Sleep disorder	3 (4)	–	–	–
Medication—neuroactive or psychoactive, no. (%)	35 (49)	–	–	–
Education in years, mean (SD)	16.3 (3.1)	–	–	–
Blood pressure, mmHg, mean (SD)	121/71 (14/9)	–	–	–
BMI, mean (SD)	26.7 (4.2)	–	–	–
AUDIT scores, mean (SD)	4.9 (6.3)	–	–	–
Time between functional tests, mean (SD)	3.0 (1.5)	–	–	–
**Functional scores, mean (SD)**				
GOS-E	6.5 (1.3)	–	–	–
NSI	16.0 (15.2)	–	–	–
SWLS	25.2 (7.7)	–	–	–
**EV concentration, mean, (SD)**				
GFAP, pg/mL	51.3 (30.6)	25.7 (15.5)	< 0.001	28.8
NfL, pg/mL	1.7 (1.2)	1.3 (0.7)	0.791	0.7

*TBI* traumatic brain injury, *HV* healthy volunteer, *BMI* body mass index, *AUDIT* Alcohol Use Disorders Identification Test, *GOS-E* Glasgow Outcome Scale-Extended, *NSI* Neurobehavioral Symptom Inventory, *SWLS* Satisfaction with Life Scale, *NfL* neurofilament light, *GFAP* glial fibrillary acidic protein, *EV* extracellular vesicle.

### Biomarker concentrations across TBI severity and compared to controls

**Figure 1:**
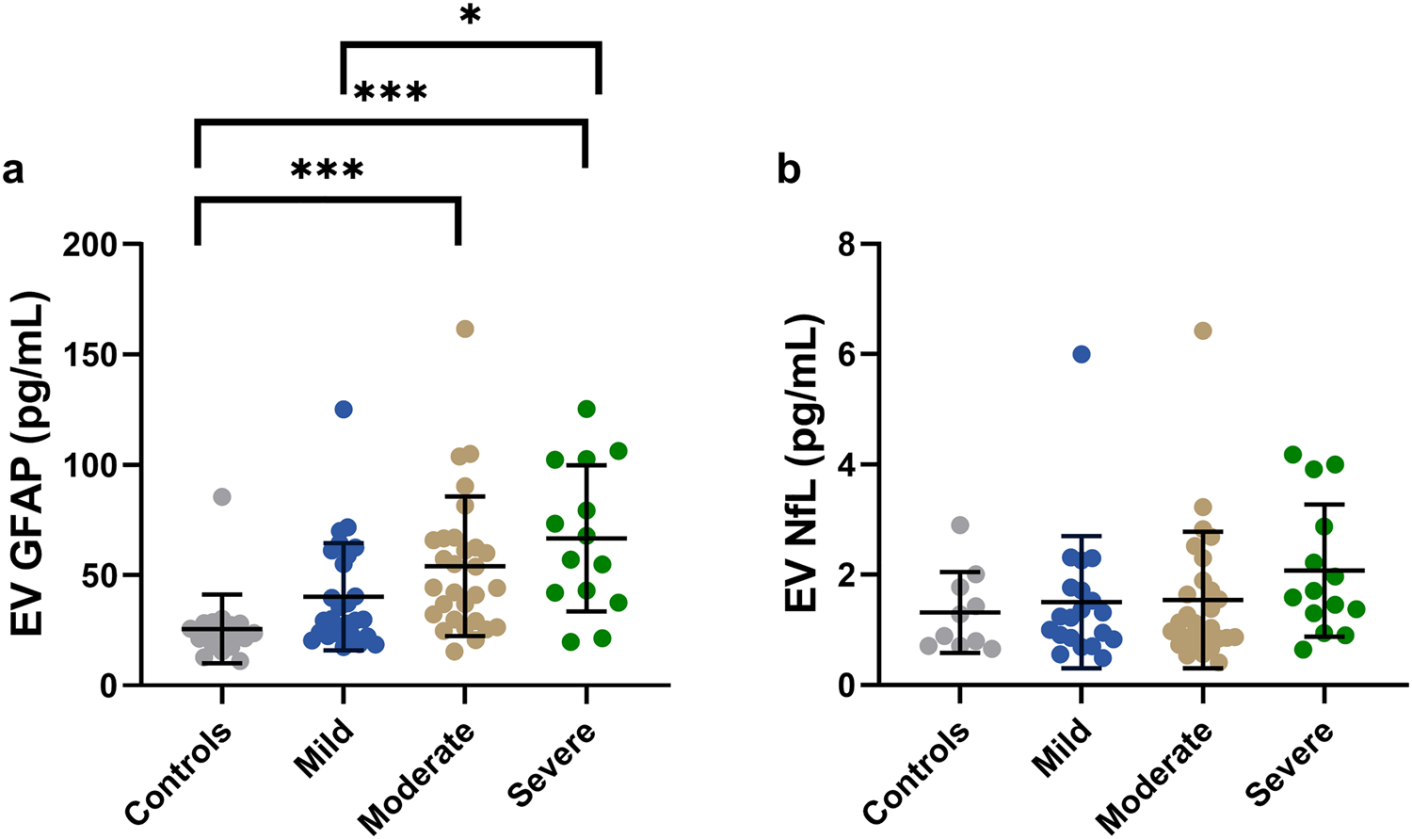
Biomarker differences in TBI patients compared to controls and across TBI severity. (**a**) EV GFAP was elevated in modTBI and sTBI compared to controls as well as in sTBI versus mTBI (n = 89). (**b**) EV NfL showed no significant alterations across TBI severity groups or between TBI severity groups and controls (n = 71). Error bars represent SD, with the middle bar indicating the mean. *p < 0.05, ***p < 0.001, adjusted for multiple comparisons. *GFAP* glial fibrillary acidic protein, *NfL* neurofilament light chain, *EV* extracellular vesicle.

EV GFAP concentrations were elevated in modTBI (*p* < 0.001, mean 54.1, SD 31.6) and sTBI (*p* < 0.001, mean 66.7, SD 33.2) compared to controls (mean 25.7, SD 15.5) as well as between sTBI and mTBI (*p* = 0.023, mean 40.2, SD 24.3, Fig. 1a). Although it did not reach statistical significance (*p* = 0.054), the mTBI group had higher mean concentrations of EV GFAP than concentrations found in the controls (Fig. 1a). EV NfL did not reach statistical significance when comparing between TBI severity groups and controls as well as across TBI severity groups (Fig. 1b).

### Biomarker diagnostic utility

**Figure 2:**
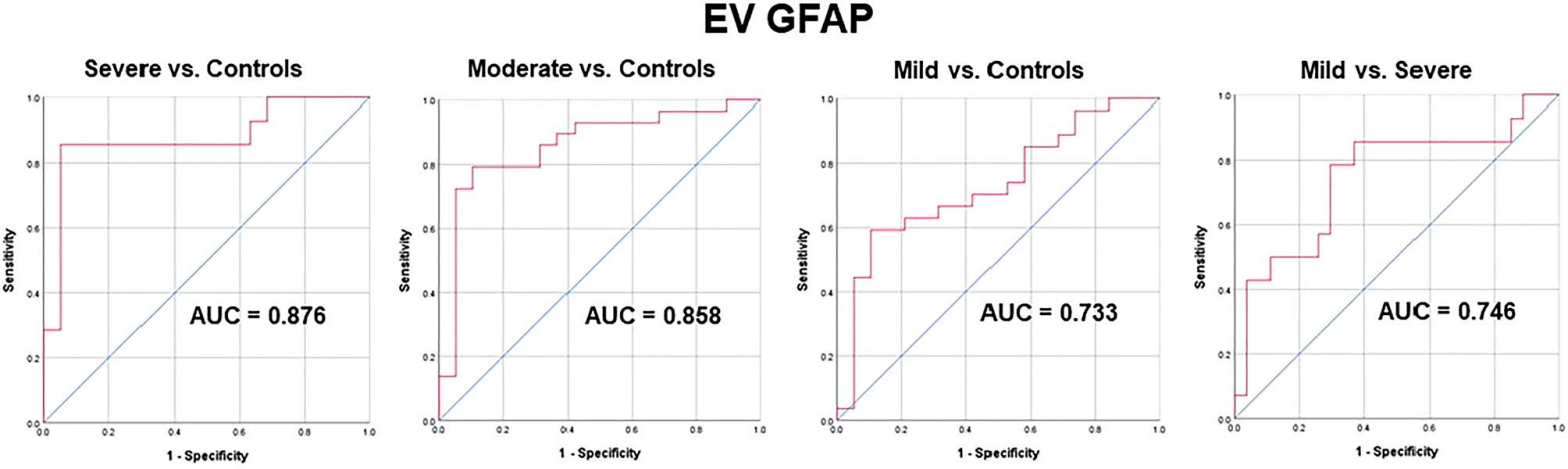
Receiver-operating characteristic curves comparing TBI severities and TBI to control. The diagonal blue line indicates the reference line (AUC = 0.5). *GFAP* glial fibrillary acidic protein, *EV* extracellular vesicle.

Area under the receiver-operating characteristic curve (AUROC) analysis was used to assess the ability of the biomarkers to discriminate between TBI patients and controls, as well as across TBI severity. Figure 2 displays the ROC AUC curves for EV GFAP, which were as follows: sTBI versus controls (0.88, 95% CI 0.74–1.0), modTBI versus controls (0.86, 95% CI 0.74–0.97), mTBI versus controls (0.73, 95% CI 0.59–0.88), mTBI versus sTBI (0.75, 95% CI 0.58–0.92). All other analyses were insignificant.

### Biomarker concentrations at 1 year and outcomes

**Figure 3:**
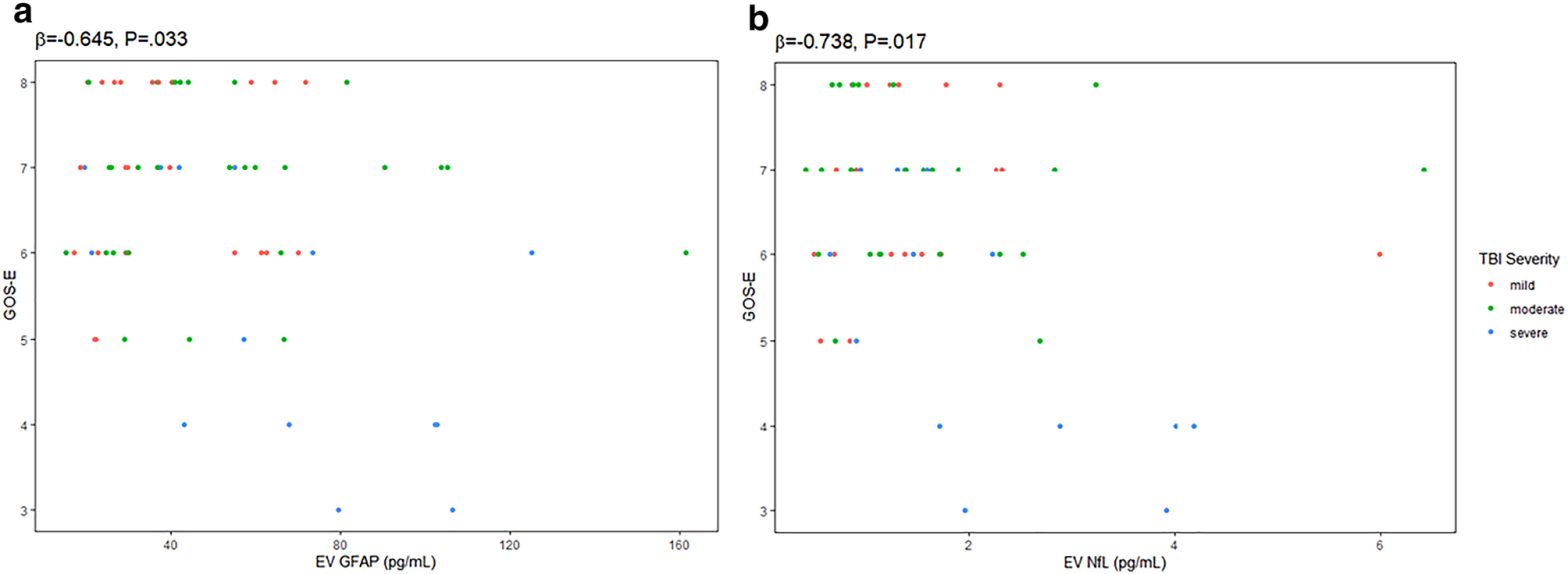
Correlations between 1 year (**a**) EV GFAP (N = 68) and (**b**) EV NfL (N = 59) concentrations and outcomes at 1 year, controlling for age and sex. *GOS-E* Glasgow Outcome Scale-Extended, *GFAP* glial fibrillary acidic protein, *NfL* neurofilament light, *EV* extracellular vesicle.

Linear regression models, controlling for age and sex, were performed to assess the relationship between biomarker concentrations and outcomes. Higher EV GFAP was associated with lower 1 year postinjury GOS-E (*β* = − 0.644, *p* = 0.033, Fig. 3). There was also an association between elevated EV NfL (*β* = − 0.738, *p* = 0.017) and lower 1 year postinjury GOS-E score (Fig. 3). No associations were found between biomarker concentrations and NSI or SWLS scores at 1 year (Table 2).

**Table 2:** Linear model of 1 year outcomes and biomarker concentrations, controlling for age and sex.

	GOS-E			NSI			SWLS		
	N	β	*p*	N	β	*p*	N	β	*p*
EV GFAP	68	− 0.644	0.033	68	− 3.166	0.386	62	− 0.528	0.7820
EV NfL	59	− 0.738	0.017	59	− 3.539	0.364	53	− 1.413	0.513

*GOS-E* Glasgow Outcome Scale-Extended, *NSI* Neurobehavioral Symptom Inventory, *SWLS* Satisfaction with Life Scale, *NfL* neurofilament light, *GFAP* glial fibrillary acidic protein, *EV* extracellular vesicle.

### Biomarker concentrations at 1 year and change in long-term outcomes

Linear regression models, controlling for age and sex, were also performed to assess the relationship between EV biomarker concentrations and change in outcomes. Higher EV GFAP (*β* = 0.604, *p* = 0.028) was associated with long-term improvement in GOS-E (Table 3). There were no significant associations between biomarker levels and SWLS or NSI score.

**Table 3:** Generalized linear model of change in outcome scores and biomarker concentrations, controlling for age and sex.

	∆GOS-E			∆NSI			∆SWLS		
	N	β	*p*	N	β	*p*	N	β	*p*
EV GFAP	48	0.604	0.028	47	1.522	0.576	44	1.695	0.410
EV NfL	44	0.389	0.112	43	− 0.185	0.936	40	3.002	0.115

*GOS-E* Glasgow Outcome Scale-Extended, *NSI* Neurobehavioral Symptom Inventory, *SWLS* Satisfaction with Life Scale SWLS, *NfL* neurofilament light, *GFAP* glial fibrillary acidic protein, *EV* extracellular vesicle.

Additional linear models controlling for outcome score at 1 year were performed post hoc. Due to low statistical power, sex and age were not controlled for in these models (sex and age had not been associated with biomarker concentrations in the previous models). We found that the previously identified relationships with change in GOS-E remained for EV GFAP (*β* = 0.435, *p* = 0.0261).

## Discussion

The main findings of this study were that EV GFAP was significantly elevated in TBI patients with a moderate or severe TBI 1 year postinjury. Further, we report that higher EV GFAP and EV NfL levels 1 year after injury correlated with worse 1 year clinical outcomes. These findings suggest that even remote injuries are associated with GFAP and NfL concentrations measured in EVs, and that this increase in activity may contribute to long-term symptoms, especially in those patients who sustained moderate or severe brain injuries.

The finding of chronic elevations of EV GFAP and NfL at 1 year postinjury in patients with moderate or severe TBIs expands on our previous work in which EV and plasma NfL levels were elevated many years after injury in military personnel with repetitive mTBI^9^. Our finding that EV GFAP and NfL continue to have diagnostic and prognostic utility when sampled at 1 year postinjury constitute increasing evidence that GFAP and NfL are valuable chronic biomarkers of TBI.

The pathophysiology underlying chronic elevations of GFAP and NfL is uncertain. Elevated GFAP is considered an indicator of astrocytic cell impairment following TBI^16,17^, and elevated NfL is suggestive of neuroaxonal damage^18–20^. Levels of NfL in exosomes and other EVs following TBI have been evaluated in few studies, with variable results^9,21–23^. A recent study has shown an association between GFAP levels in EVs, but not free-circulating GFAP, and presence of diffuse injury as compared to other lesion types^23^. Additional studies are needed to elucidate the mechanisms leading to chronic elevations of these biomarkers and to determine their effects.

We found that increased EV GFAP at 1 year following TBI was associated with improvement in GOS-E up to 5 years postinjury. This is contradictory to previous research in which higher markers of neurodegeneration were associated with poorer outcomes^8,24^. However, a study on sports-related concussion found that higher plasma levels of GFAP were associated with better recovery after injury^25^. Additional research is needed to understand these discrepancies. In our cohort, we did not control for the treatment patients may have received, and it is possible that individuals with higher neurodegeneration were more likely to seek treatment and thus report greater improvements. EVs in preclinical studies of neurological disorders have been shown to have a complex relationship with disease progression, at times promoting unhealthy protein aggregation^26^ and at times promoting clearance^27,28^. Our finding adds to this complex picture of how proteins carried in EVs may impact disease progression or recovery.

There were several limitations to this study. First, our sample size was relatively small, and all blood draws were at 1 year postinjury with variable timing of testing for final outcome scores. Second, we did not have longitudinal EV measurements, restricting us from assessing the temporal profiles of EV GFAP and NfL. Lastly, our TBI cohort had significantly more white patients and fewer African American patients compared to the control cohort. Future research investigating EV biomarkers in larger cohorts and multiple time points following TBI is needed to confirm our findings that EV GFAP and NfL may be useful chronic biomarkers for TBI.

In conclusion, EV GFAP remained elevated at 1 year postinjury in TBI patients. EV GFAP, as well as EV NfL, measured at 1 year postinjury were associated with long-term outcomes in TBI. EV GFAP had particularly strong discriminatory ability between TBI patients and controls and was associated with outcomes, suggesting that EV biomarkers may have clinical relevance, and that further investigation into EV blood-based biomarkers for TBI is warranted.

## Methods

### Study population

This analysis is part of the Long-term Clinical Correlates of Traumatic Brain Injury study (NCT01132898, 05/28/2010), an ongoing longitudinal, natural history study of TBI at the National Institutes of Health. The methods of this study have been described previously^10,11^.

Inclusion criteria required that patients were (i) 18 years or older and could speak and read English, (ii) diagnosed with a non-penetrating TBI, and (iii) enrolled within 1 year of their injury. Exclusion criteria were (i) pregnancy, (ii) contraindication to MRI, and (iii) history of significant psychiatric or neurologic conditions. TBI classification was determined per the Veterans Affairs/Department of Defense severity rating scale applied by a clinician who was blinded to patient biomarker levels^29^. All patients had blood drawn at 1 year postinjury and outcome scales administered annually beginning at 1 year postinjury. Eight patients had a final visit time point at 2 years postinjury, 12 at 3 years, 11 at 4 years, 20 at 5 years, and 21 did not have a follow-up visit. Biomarkers were also quantified from 20 healthy volunteers from another NIH study (NCT00888563, 04/27/2009). Inclusion criteria for healthy controls were: (i) 18 years of age or older and could speak and read English, (ii) good general medical and psychological health based on History and Physical by licensed medical staff, (iii) no history of heavy alcohol use or substance abuse, (iv) no history of a disease or condition that causes significant fatigue (congestive heart failure, cancer, or sleep disorders) or a history of taking medicines that cause fatigue (beta blockers, diuretics, or narcotics), and (v) no history of head injury. All participants provided written informed consent to participate. The study was approved by and followed the ethical standards of the NIH institutional review board, and all methods were performed in accordance with guidelines and regulations. Participants also provided written consent for blood to be drawn, stored, and analyzed.

### Functional outcomes

The Glasgow Outcome Scale-Extended (GOS-E), Neurobehavioral Symptom Inventory (NSI), and Satisfaction with Life Scale (SWLS) were administered by a qualified researcher at each time point. Change in outcome score ($\Delta )$ was calculated by subtracting the patient’s score at 1 year from the patient’s score at the final visit timepoint. The GOS-E is a clinician-rated measure that assesses overall function after head injury on a 1–8 scale, where 1 corresponds to death and 8 to upper good recovery^30,31^. It is widely used as a primary outcome measure for head injury and has been demonstrated to be valid and reliable^32^. The NSI assesses self-reported postconcussive symptoms on a scale of 0–88 with higher scores corresponding to increased symptom burden^33^. It is commonly used, including by the Department of Defense and Veterans Affairs, and has high internal consistency (r = 0.95)^34^. The SWLS assesses a patient’s global life satisfaction on a scale ranging from 5 to 35, with a score of 35 corresponding to very high life satisfaction, and has been shown to be a valid and reliable measure of life satisfaction^35,36^.

### Biological samples

Blood samples were collected via venipuncture into gel-separator tubes for serum, centrifuged, and stored at − 80 °C. Prior to EV isolation, samples were processed according to International Society of Extracellular Vesicles (ISEV) guidelines^37^. Time to centrifugation and storage was variable between approximately 45 min to 2 h. Researchers were blinded to patient clinical information throughout blood processing, EV isolation, and protein measurement.

### EV isolation

We have previously reported our method for EV isolation^14^. In brief, EVs were isolated using ExoQuick exosome solution (System Biosciences, Inc., Mountain View, CA, USA) according to manufacturer instructions. To lyse EVs, equal volume of mammalian protein extraction reagent (M-PER) was added (Thermo-Fisher Scientific, Inc., Rockford, IL, USA). After EV isolation and lysis, samples were stored at − 80 °C until quantification of each analyte. EV samples were eventually eluted in their original volume of serum. Therefore, all the expressed values refer to the original serum volume.

### Protein measurement

We measured EV levels of GFAP, NfL, ubiquitin c-terminal hydrolase L1 (UCH-L1) and tau in duplicate using Simoa (Quanterix, Lexington, MA) on a High-Definition-1 Analyzer with the Simoa Neuro 4-Plex Advantage Kit (Quanterix, Lexington, MA; cat 102153). Inter-plate variations were less than 10% for all samples, and reported coefficient of variations (CVs) for individual samples were acceptable if they were under 25%. Measurements of EV UCH-L1 and EV tau were either undetectable or had CVs above the 25% cutoff in more than 20% of the samples, and thus data were not of sufficient quality to be included in the analyses. EV GFAP had one TBI patient and one healthy control value with a CV over 25%. EV NfL had the larger number of values with CVs > 25%: 10 TBI patients and 10 controls. The average CVs for the EV GFAP samples were 3.4%, and for EV NfL were 10.1%, respectively. The limit of detection for the assay was 0.22 pg/mL for GFAP and 0.10 pg/mL for NfL.

### Statistical analyses

Data analyses were conducted using R (v. 3.6.3, The R foundation for Statistical Computing, Vienna, Austria), IBM SPSS Statistics (Version 26), and GraphPad Prism 8.2.0. Biomarker concentrations were not normally distributed; as such, these values were natural log transformed for all analyses and comparisons. For clarity, figures are presented and results are described using the original, untransformed data. One-way ANOVA tests with Bonferroni correction and chi-squared tests were performed to determine group differences in demographic characteristics between TBI patients and controls. One-way ANOVA tests with Bonferroni correction were performed to determine group differences in biomarkers across TBI severities and between TBI severities and controls. Receiver-operator characteristic (ROC) area under the curve (AUC) analyses were performed to assess biomarker performance. Multivariable linear regression models covaried for age and sex were constructed to determine the association between each biomarker and functional outcome score at 1 year postinjury and with change in outcome scores from 1 year to final time point. All tests were two-sided and statistical significance was set at *p* < 0.05.

One patient with a chronic autoimmune disease had extremely high concentrations for EV samples, between 10 and 1000 times higher than any other patient. Analyses were conducted with and without this individual and it was found that this patient significantly influenced statistical outcomes; therefore, this patient was removed from all analyses.

## Data Availability

The data that support the findings of this study are available upon reasonable request to the corresponding author.
